# Developmental Features, Influencing Factors, and Formation Mechanism of Underground Mining–Induced Ground Fissure Disasters in China: A Review

**DOI:** 10.3390/ijerph20043511

**Published:** 2023-02-16

**Authors:** Yu Li, Hui Liu, Lijuan Su, Sidi Chen, Xiaojun Zhu, Pengfei Zhang

**Affiliations:** 1 School of Resources and Environmental Engineering, Anhui University, Hefei 230601, China; 2 Anhui Province Engineering Laboratory for Mine Ecological Remediation, Hefei 230601, China; 3 School of Mathematical Sciences, Anhui University, Hefei 230601, China; 4 School of Environment and Spatial Informatics, China University of Mining and Technology, Xuzhou 221116, China

**Keywords:** ground fissure, rock fracture, surface subsidence, coal mining, eco–environment protection

## Abstract

Mining–induced ground fissures are one of the major geological disasters affecting coal mines. In recent years, many effective monitoring methods have been developed to explore the developmental characteristics and nature of mining–induced ground fissures for being treated scientifically. This paper is mainly on the development law and mechanism of mining ground fissure research results which have been comprehensively combed, highlighting the development trend, including the formation condition, development features, influencing factors, and mechanical mechanism of mining–induced ground fissures. Outstanding issues are discussed and future research hot spots and trends are pointed out. The major conclusions include: (1) under the shallow coal mining condition, because the rock layer fault zone directly reaches the surface, the ground fissure usually develops seriously; (2) mining–induced ground fissures are generally divided into four types: tensile fissures, compression fissures, collapsed fissures, and sliding fissures; (3) mining–induced ground fissures are affected by the coupling effect of underground mining and surface topography. The main factors are geological mining conditions, surface deformation, and surface topography, including rock and soil structure, rock and soil mechanical properties, surface horizontal deformation, surface slope, and so on; and (4) to ensure the safety of underground mining, temporary ground fissures formed during the process of coal mining must be treated when ground fissures and rock ground fissures are connected. The results of this article make up for the deficiencies of the relevant research, provide the basis and direction for future research, and have universal applicability and scientific guiding significance.

## 1. Introduction

Coal will remain the world’s main energy source for a long time to come. However, in the past few hundred years, underground coal mining has caused a series of environmental problems such as land destruction, air pollution, and coal mine waste accumulation [1]. Among these man–made disasters, ground fissure disasters caused by mining have attracted increasingly more attention due to them leading to serious environmental damages such as land loss, soil erosion, and vegetation withering [2]. Mining–induced ground fissures are a typical form of nonlinear mechanical disaster in coal mining areas. They are the result of the coupling between the movement of the overlying strata and the deformation of the topsoil in the goaf after coal mining [3]. The process of formation of ground fissures is a complex mechanical process. The width and depth of ground fissures are closely related to the presence or absence of Quaternary loose layers and their thickness, physical and mechanical properties, and critical deformation values [4]. Mining induces the occurrence of ground fissures and brings a series of problems. The hazards are mainly manifested in the following aspects. (1) Exacerbating land damage. Ground fissures usually occur simultaneously with surface subsidence. Therefore, once ground fissures form in a surface subsidence basin, the soil is destroyed, resulting in the discontinuous deformation of the surface. These fissures are often difficult to repair, and land that could have been cultivated becomes fragmented. (2) Soil erosion intensifies. Once ground fissures occur on the surface, it is difficult to maintain the water in the aquifer in the soil; a large amount of surface water will flow away through the ground fissures, and a large amount of water in the soil will evaporate, resulting in a decline in the soil’s quality. (3) Destruction of surface vegetation. Soil ground fissuring can damage the root system of plants, making it difficult for plants to obtain the nutrients they need to grow through the root system; the result is that a large number of plants wither or die. (4) Surface ecosystems are destroyed. Whether in arid areas or water–rich areas, ground fissure disasters destroy the original balanced ecosystem. Plants die because of a lack of nutrition, herbivores migrate because they cannot find edible food, and the damage to ecosystems caused by ground fissures is difficult to repair [3,5,6].

In recent years, with the implementation of China’s coal strategy, ground fissure disasters in mining areas caused by mining activity have become increasingly serious. Based on rock movement and surface deformation research, studies on rock fracture and ground fissure disasters caused by mining have gradually become more detailed [7]. According to the available literature, Chinese scholars have paid attention to the problem of mining–induced ground fissures in coal mining areas since the 1990s. Kang Jianrong (2008) analyzed the four stages of mining fractures and their formation process based on field measurement data and revealed the influence of mining fractures on the movement and deformation of mountainous surfaces [8,9]. Zhu Guohong et al. (2012) analyzed the cumulative effect of mining–induced surface fractures in a mining area based on mining subsidence and rock mechanics theory. The probability integration method, Hooke’s law, and Mohr–Coulomb failure criterion have been used to describe and quantify the characterization index of the mining–induced cumulative effect of surface tensile fractures. Characterization indexes of the time crowding effect and the mining delay effect of mining fractures and the characterization indexes of the mining space crowding effect have been put forward [10]. Zheng Hui et al. (2011) obtained the angular parameters for determining the distribution range of fractures and the dynamic development process of fractures, and analyzed the relationship between fractures and horizontal deformation through a study of the plane distribution of mining–induced ground fissures [11]. Liu Donglin, Xu Jialin et al. (2012), and others analyzed the difference in mountain fractures when mining on the working face slope and downhill through UDEC numerical simulation. They concluded that the development of fractures is large during downslope mining, and push–type reverse fractures easily form [12].

Since the 21st century, the development of ground fissures in China has continued to increase, being manifested in the continuous extension of existing ground fissures and the continuous emergence of new ones [13]. By 2015, more than 5000 ground fissures had been discovered in more than 4000 places in 25 provinces and 3 municipalities directly under the Central Government. Ground fissures in China are mainly distributed in the Fenwei Basin, the Hebei Plain, and the Yangtze River Delta [14]. These ground fissures are also the most serious, and the economic losses caused by them account for about 80% of the total economic losses [14]. To reduce or control the damage from ground fissures to the ecological environment of a mining area, first, a variety of technical means are used to monitor them, including traditional methods based on field measurements, methods based on large–scale monitoring, and satellite remote sensing technology [15]. The advantages of on–site monitoring are high precision and accurate positioning, but the disadvantage is that the on–site work intensity is high. In contrast, although remote sensing technology can be used to obtain ground fissure information efficiently and quickly, it is often limited by positioning accuracy, resulting in the loss of small fissure information [16]. Secondly, the development characteristics and behavior of mining–induced ground fissures are studied. On this basis, combined with underground mining data, the main factors affecting the development of ground fissures are determined, and an empirical model between geological mining conditions and ground fissures is established. In addition, by studying the mechanical characteristics of formation movement and surface deformation, a mechanical model of mining–induced ground fissures has been established [17]. Finally, attempts have been made to control or manage ground fissures, and methods such as caulking, reclamation of subsidence, and re–vegetation have been developed. Overall, recent developments in mining–induced ground fissure hazards research show an evolution from monitoring techniques to treatment methods [18]. However, under different geological and mining conditions, how ground fissures form is still not fully understood. Are their mechanisms the same? What are the key scientific questions needed to solve the problem of ground fissure management? These problems directly affect the prediction and prevention of ground fissure disasters. Therefore, it is necessary to conduct a comprehensive review of existing mining–induced ground fissure disaster research from a systems perspective, which is the main goal of this study.

The rest of the paper is organized as follows: Section 2 presents a survey of mining–induced ground fissures in China; Section 3 describes what mining–induced ground fissures are; Section 4 comprehensively reviews the influencing factors of mining–induced ground fissures; then, Section 5 provides information on the formation mechanism of mining–induced ground fissures; Section 6 discusses how to deal with ground fissures caused by mining; Section 7 presents open issues; and Section 8 draws conclusions.

## 2. Investigation of Mining–Induced Ground Fissures in China

**Figure 1:**
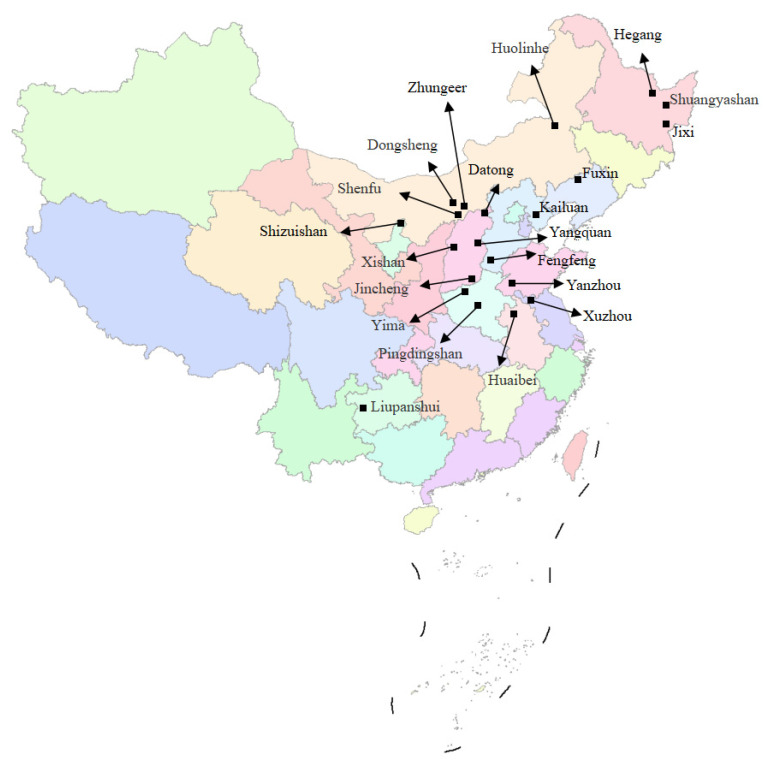
The distribution of mining–induced ground fissures in China.

**Figure 2:**
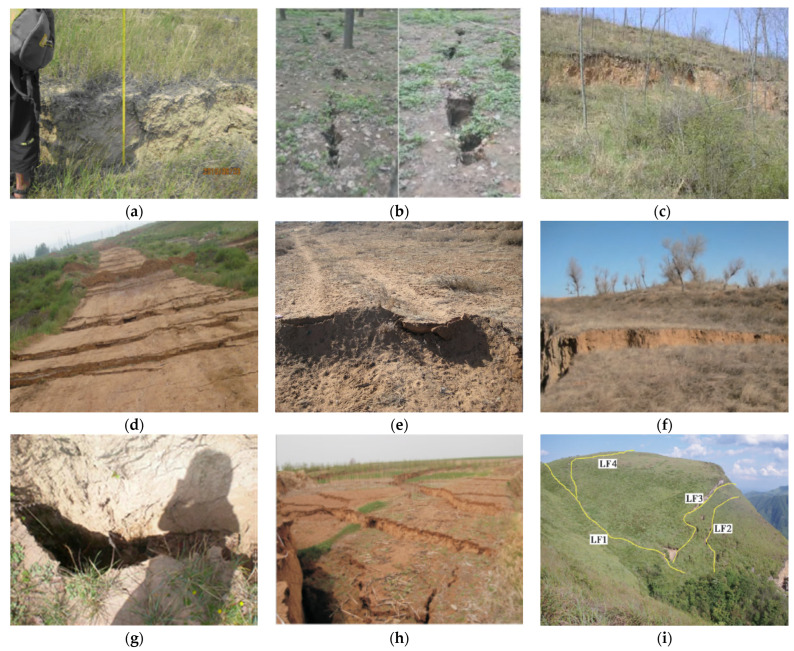
Mining ground fissure disasters in typical mining areas in China. (**a**) Huainan 8104 (Collapse fissure); (**b**) Pingdingshan 11022 (Stretch fissure); (**c**) Tongchuan D508 (Sliding fissure); (**d**) Shendong 12208 (Collapse fissure); (**e**) Shendong 22201 (Squeeze fissure); (**f**) Shendong 52304 (Sliding fissure); (**g**) Binchang B40301 (Stretch fissure); (**h**) Hezhai 2212 (Collapse fissure); (**i**) Jieniangping (Collapse and Sliding fissures).

As the third–largest coal producer in the world, China has a total coal resource of 5.9 trillion tons. Since large–scale mechanized coal mining was implemented in 1980, 1.4 billion–ton large–scale coal production bases have been formed. In 2018, China’s total coal production was 3.546 billion tons, which was about half of the world’s total coal production [19]. The mining of coal resources has resulted in a large number of geological disasters, including ground collapse, ground subsidence, ground fissures, and landslides. We counted the ground fissure disasters caused by coal mining in 21 mining areas in China from 1997 to 2022, involving 10 major coal–producing provinces, as shown in Figure 1. Among them, three different geomorphological conditions are included: plain areas (Shandong, Anhui, Hebei, and Liaoning), the Loess Plateau (Gansu and Inner Mongolia), and hilly areas (Shaanxi, Shanxi, Guizhou, and Henan) [20]. Development information on the ground fissures is shown in Table 1, and field pictures of some ground fissures are shown in Figure 2.

From the perspective of geological mining conditions, most of the mining areas in eastern China have the characteristics of deep burial, complex geological structure, and flat terrain. However, the western mining area has the characteristics of thick coal seams, shallow burial, and large terrain fluctuations [36]. Combined with the data, we can see that the mining ground fissure disaster is mainly medium or shallow coal seam, and the mining of shallow coal seams is more likely to cause ground fissures. The characteristics and causes of mining–induced ground fissures are also different. Among them, the surface deformation caused by mining is the main cause of the development of ground fissures (Figure 2b,e,g); further rock fractures most typically occur during shallow coal mining (Figure 2a,d,h), and these two inducements are more common in plain areas. In mountainous or hilly areas, mining areas are prone to landslides, leading to the formation of ground fissures ((c),(f)). In faulted mining areas, mining can also cause fault activation to generate ground fissures on the surface (i) [37]. Surface stretching and strata breaking are the main reasons for the development of mining–induced ground fissures, while slope slip and fault activation induce the further expansion of ground fissures. The mining of deeply buried coal seams is dominated by small tensile fractures, while the mining of shallowly buried coal seams is mainly manifested as stepped surface subsidence. However, mining under hillside terrain is likely to cause slope slippage and rupture. When there are structures such as faults in the ground, large–scale ground fissures caused by fault activation will also occur [38]. 

## 3. What Is a Mining–Induced Ground Fissure?

### 3.1. Development Process of Mining–Induced Ground Fissures

A mining–induced ground fissure refers to a kind of geological disaster caused by the ground fissuring and subsidence of the ground surface induced by the mining of underground coal resources. Mining–induced ground fissures are produced at the same time as the surface subsidence and are the result of the coupled deformation of the overlying rock layer and the topsoil layer in the goaf. The general steps for ground fissure generation are first, the mining of the longwall face causes the breaking and movement of the roof strata, the main manifestations being rock breakage, caving, fissures, etc. Rock movement zones can be divided into the caving zone, fracture zone, and bending subsidence zone from bottom to top. Next, these deformations gradually transfer to the surface, and, thus, a subsidence basin forms on the surface, which consists of three areas: tension area, compression area, and neutral area [39]. As a result, ground fissures occur. In the mining of deep coal seams, the characteristics of the “three zones” are more obvious, and the surface is mostly composed of tensile fractures. That is to say, these ground fissures are formed due to the tensile deformation of the surface caused by the force of coal mining exceeding the tensile strength of the topsoil. In shallow coal seam mining, because the fissure zone of the strata may reach the surface directly, that is to say, there is no bending subsidence zone, the surface will not only produce tension ground fissures in the tension zone but also form step–like collapse ground fissures above the goaf. Therefore, the formation of mining–induced ground fissures can be divided into three stages: formation and expansion of rock fractures, surface deformation and fissures formation, and dynamic development of ground fissures [40].

#### 3.1.1. Formation and Expansion of Rock Fractures

**Figure 3:**
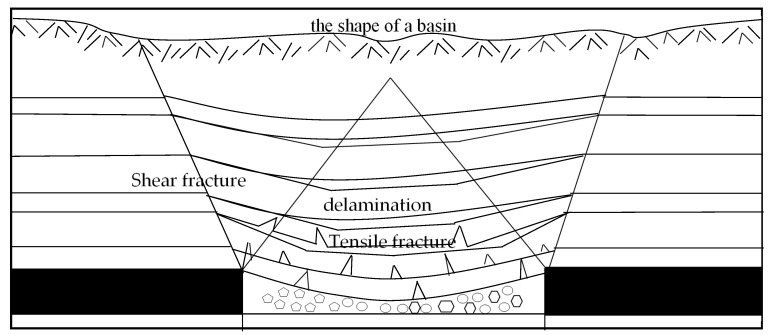
Evolution diagram of rock fractures.

In the early stage of mining, the original stress balance of the overburden rock is broken, and the roof rock gradually bends downward. When the overburden stress exceeds the strength of the roof rock, the roof of the rock stratum breaks for the first time, and the rocks cave and fill the goaf. Next, with the area of the direct roof hanging increasing, fine ground fissures are generated inside the rock stratum and extend and diffuse around it. When the ground fissures accumulate to a certain extent, the rock stratum breaks off, and the strata with different lithologies will form a transverse distribution of the separation layer. At the same time, the vertical ground fissures on the top of the goaf extend continuously from bottom to top, with the boundary on both sides of the goaf acting as the starting point, and they gradually extend to the rock strata with a fracture angle of the rock generally being 45–60 degrees [41,42,43]. This process is called the formation and expansion stage of the rock fracture, as shown in Figure 3.

#### 3.1.2. Surface Deformation and Ground Fissure Formation Stage

**Figure 4:**
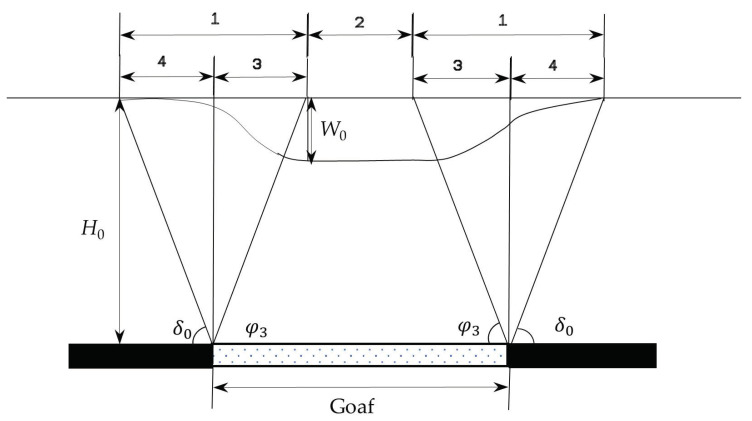
Section of the subsidence basin. *W*_0_—maximum sinking value; *H*_0_—average mining height; $\delta_{0}$—boundary angle; $\varphi_{3}$—full mining angle; 1—move the deformation zone; 2—uniform subsidence area; 3—compression deformation zone; 4—stretch deformation zone.

When the rock layer moves and transfers the surface, the surface moves and deforms, so that a subsidence basin with a much larger area than the goaf is formed on the surface above the goaf. According to the characteristics of surface deformation, surface mobile basins can be divided into a tensile deformation area, compression deformation area, and uniform subsidence area. Among them, the tensile zone and the compression zone take the inflection point of the surface deformation as the dividing point, and the uniform subsidence zone refers to the area between the two maximum subsidence points, as shown in Figure 4. In the tensile deformation zone, when the horizontal deformation value of the surface exceeds the tensile strength of the topsoil, surface tensile ground fissures are formed. In the compressive deformation area, if the compressive deformation of the ground surface is greater than the compressive strength of the topsoil, uplift–shaped ground fissures form. In the uniform subsidence area, the horizontal deformation of the surface is 0, and generally, no ground fissures develop [26,44].

#### 3.1.3. Dynamic Development Stage of Ground Fissures

**Figure 5:**
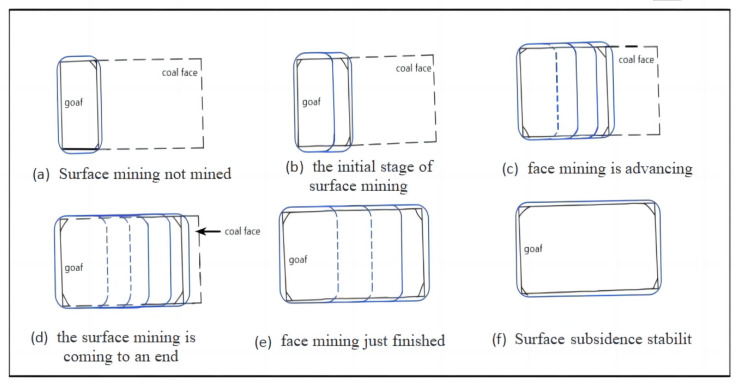
Dynamic development process of surface fractures.

Ground fissures can be divided into two categories according to their location: temporary fissures just above the goaf and permanent fissures on the boundary. Temporary fractures generally occur just above the working face and are periodic, that is to say, the development of ground fissures undergoes a complete “ground fissure and closure” cycle process [45], as shown in Figure 5.

**Figure 6:**
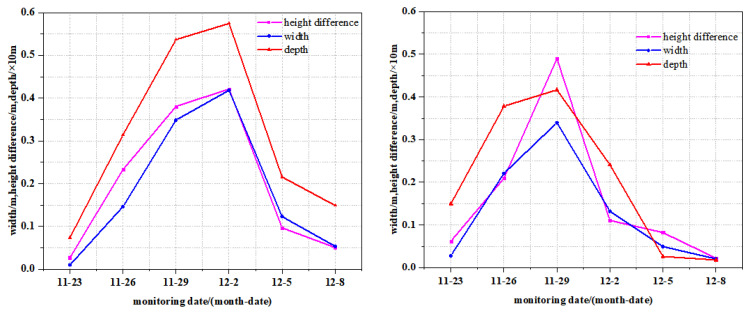
The relationship between the width, drop, depth, and time of ground fissures.

**Figure 7:**
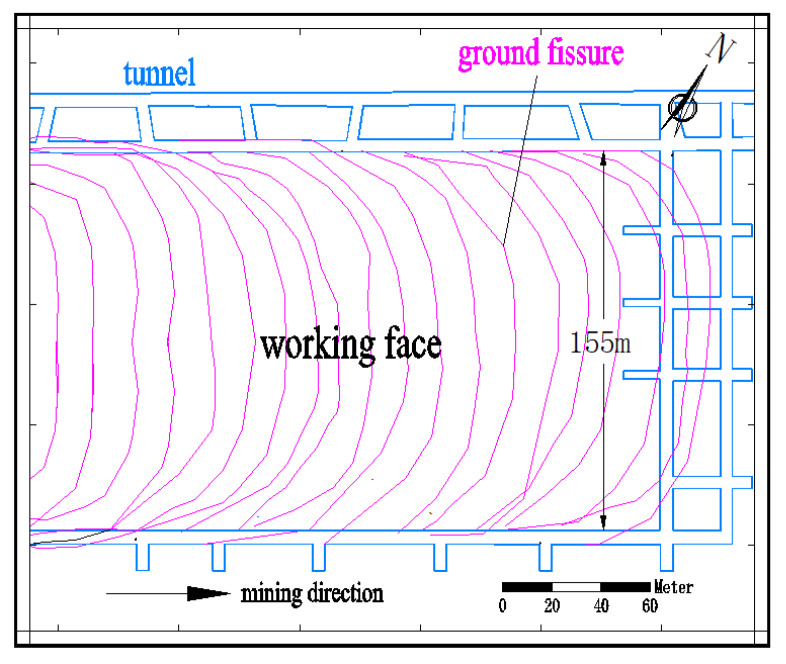
Schematic diagram of the ground fissure and corresponding working face sections during mining. (The pink line represents the ground fissure measured in the previous coal seam, and the blue line represents the face tunnel).

The dynamic development process is as follows: in the early stage of mining, ground fissures are generated with the periodic breaking of the rock formation, and they continue to expand. Next, with the continuous advancement of the working face, the movement of the rock strata behind reaches a new balance, the surface mobile basin continues to expand forward, and the horizontal deformation in the central area gradually becomes 0. Therefore, the ground fissures are also gradually healed, and such ground fissures are called temporary fissures. For example, during the mining of the Daliuta Coal Mine, the width, drop, and depth of the two ground fissures first increased and then decreased with time, and finally healed, as shown in Figure 6. Permanent ground fissures generally occur at the boundary of the goaf. When mining is completed, the surface tensile deformation at the boundary of the working face reaches the maximum value [46,47]. From the beginning to the end of mining, the ground fissures gradually increase and cannot heal themselves, and will permanently exist on the surface, as shown in Figure 7.

### 3.2. General Characteristics of the Development of Mining–Induced Ground Fissures

The development characteristics of mining–induced ground fissures have always been a research hot spot. Many scholars have carried out many studies through field measurements, remote sensing monitoring, laboratory simulations, and other methods, and have obtained the following common characteristics. In the early stage of mining, when the surface tensile deformation is enough to cause the topsoil to break, the first mining ground fissure appears, which initially appears on the surface above the center of the panel and perpendicular to the direction of advancement, and then extends to the boundary of the goaf [48]; the ground fissures are widest and deepest in the middle and gradually narrow and become shallower as they approach the boundary. The average spacing of the fractures is consistent with the periodic breaking step of the basic top of the rock formation; with the advancement of the working face, the width and drop of the rear ground fissure gradually decrease and heal within a period of time after the surface subsidence stabilizes. At the same time, the ground fissures in front repeat the development process; all dynamic fractures present a “C”–shaped plane distribution, and the fracture range is close to or slightly beyond the boundary of the gob. After mining, the fractures at the boundary of the goaf do not close, showing an elliptical plane distribution characteristic, and the fractures at the positions of the opening and the final mining line are approximately symmetrically distributed. It can be seen that the mining–induced ground fissures have self–healing characteristics and generally heal within a period of time after mining [49]. However, after the surface subsidence is stabilized, the ground fissures located in the surface tensile deformation zone cannot be closed due to the maximum surface deformation.

### 3.3. Types of Mining–Induced Ground Fissures

According to the mechanical causes of mining ground fractures, they are mainly classified as follows:

According to the formation mechanism, mining–induced ground fissures can be divided into four types: tensile fractures, extrusion fractures, collapsed fractures, and sliding fractures, among which collapse–type fractures include surface collapse caused by rock breakage and surface collapse caused by mining–induced fault activation [40].

Tensile ground fissures are formed when the tensile deformation of the surface exceeds the tensile strength of the topsoil; its main features are lateral ground fissuring, small width, small depth, no steps on the surface, and development ahead of the mining position of the working face (as shown in Figure 2b,g). Extrusion ground fissures are the uplifts formed by the compression of the topsoil when the surface compression deformation exceeds the compression resistance of the topsoil during the mining process, and they develop in the surface compression deformation area. Some main characteristics of an extrusion ground fissure are, with the advancement of the working face, it gradually heals, the surface is convex, the ground fissure width is small, and it has a certain self–healing ability, as shown in Figure 2e. Collapsing ground fissures are caused by the overall collapse of the overlying rock and topsoil caused by the failure of the basic roof, as shown in Figure 2a,d,h or the surface collapse caused by the activation of faults caused by mining, as shown in Figure 2i. Generally, collapsed ground fissures lag behind the mining position of the working face. Their main features are horizontal ground fissuring and vertical subsidence, large width, large depth (even directly to the goaf), steps on the surface, and a development lag or synchronicity with the mining position of the working face. Generally speaking, in the process of mining, if there are collapse–type fractures on the surface, the tensile–type fractures will also develop in the tensile deformation zone of the surface in front of the goaf. Sliding–type ground fissures are formed when the working face is located under the gully terrain, and the mining easily causes the slip of the surface slope and local rupture occurs. Their main features are the formation of steps, large width, and a larger drop as shown in (Figure 2c,f,i).

From the formation process of the ground fissure, the first thing is that underground mining leads to the extension of rock fissures from the bottom up, so it leads to the generation of the ground fissure. However, it has also been suggested that topsoil cracks are caused by tensile deformation of the surface, with fissures extending downward from the surface. [50]. In fact, these two conclusions are not contradictory, the former is for the collapse ground fissure, the latter refers to the tensile ground fissure.

### 3.4. Several Characteristic Parameters Describing Mining–Induced Ground Fissures

**Figure 8:**
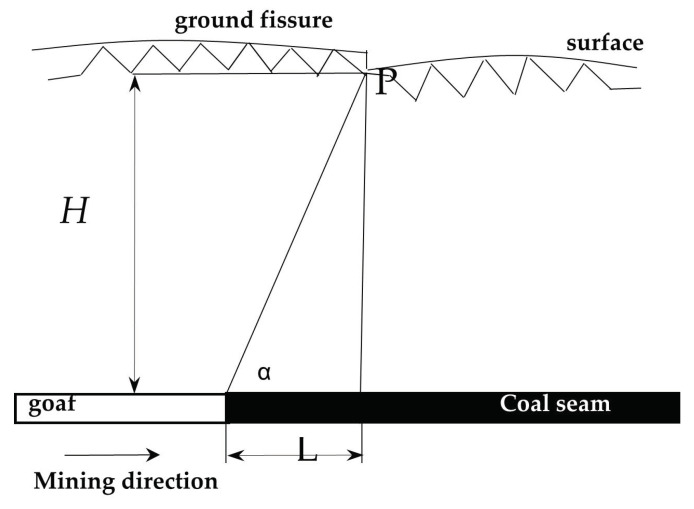
Panel outline of the ground fissure.

Generally, the size of ground fissures is described in terms of the width, depth, and drop of the ground fissures, and the development period is used to describe the duration of the dynamic development of the ground fissures. Furthermore, to further illustrate how ground fissures develop, we introduce two basic concepts [51]. *L* is the horizontal distance from the ground fissure to the goaf boundary in the horizontal direction. This is positive when the ground fissure is ahead of the advancing position and negative when the ground fissure is behind. The included angle *α* between the line connecting the fissure and the gob boundary and the horizontal line is shown in Figure 8.

The relationship between *L* and *α* is

(1) tanα=H/L

where *α* is the angle of the ground fissure, (°), *H* is the mining depth (m), and *L* is the horizontal distance of the ground fissure (m). The side biased toward the coal pillar is positive, and the side biased toward the gob is negative.

## 4. Influencing Factors of Mining–Induced Ground Fissures

Mining–induced ground fissures are formed due to the overall deformation of the rock and soil mass caused by mining, and their development is mainly affected by three factors: geological mining conditions, surface deformation, and topography. Underground coal mining is the direct cause of ground fissures. Therefore, geological mining conditions have the greatest impact on the development of ground fissures, and the main parameters include mining depth, mining thickness, rock and soil structure and lithology, mining speed, and geological structure, among others [52]. Surface deformation mainly affects the size of ground fissures, and topography mainly affects the development position of ground fissures. 

### 4.1. Influence of Geological Mining Conditions on the Development Characteristics of Ground Fissures

#### 4.1.1. Influence of Bedrock Mining Thickness Ratio on the Development of Ground Fissures

Generally, the smaller the mining depth, the thicker the coal seam, and the easier it is to form ground fissures. Therefore, in conventional geological mining, the ratio of mining depth to a mine height of 30 is generally considered to be the critical value for the discontinuity of ground deformation. The entire overburden above the coal mining area is generally considered to be composed of loose layers and bedrock layers [53]. For shallow coal seams, the tensile strength of the loose layer is weak and can often be ignored. Therefore, the fracture of the bedrock can cause the ground–to–ground fissure at the same time. Bedrock thickness, rather than mining depth, is considered a key factor in the formation of mining–induced formation fractures in shallow coal seams. For example, in the Daliuta coal mine in the Shendong mining area, many ground fissures developed during the mining of the 1–2, 2–2, and 5–2 coal seams. The average bedrock mining thickness ratios of the three coal seams are 4.5, 15.3, and 29.2, respectively. The smaller the mining–thickness ratio of the bedrock, the more obvious the discontinuous deformation of the surface, and the more fully developed the ground fissures. 

#### 4.1.2. Influence of Mining Speed on the Development Location and Period of Ground Fissures

**Figure 9:**
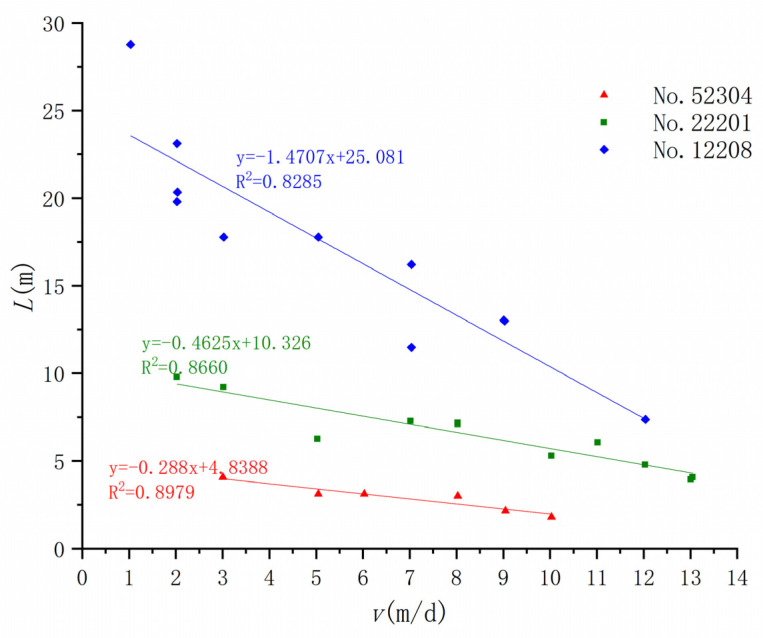
Relationship between ground fissure advance rate *v* and horizontal distance *L*.

**Figure 10:**
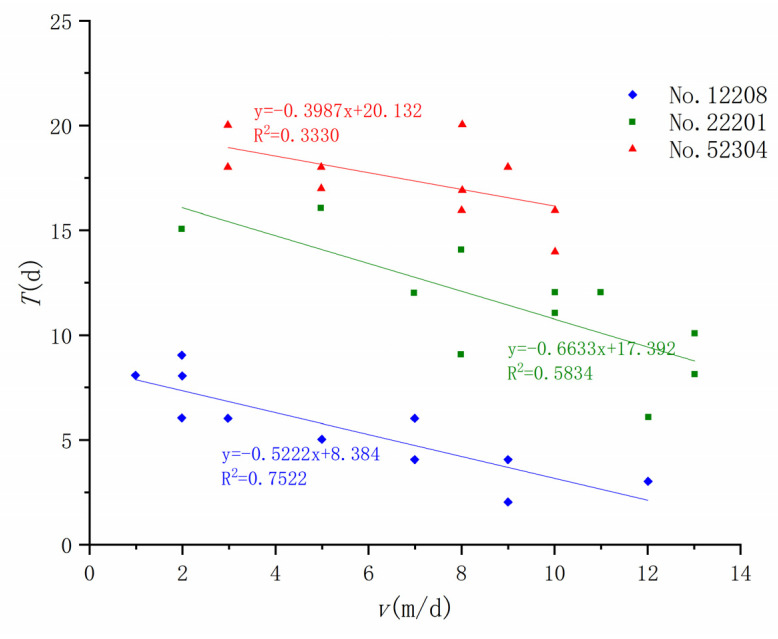
Relationship between ground fissure advance rate *v* and cycle time *T*.

**Figure 11:**
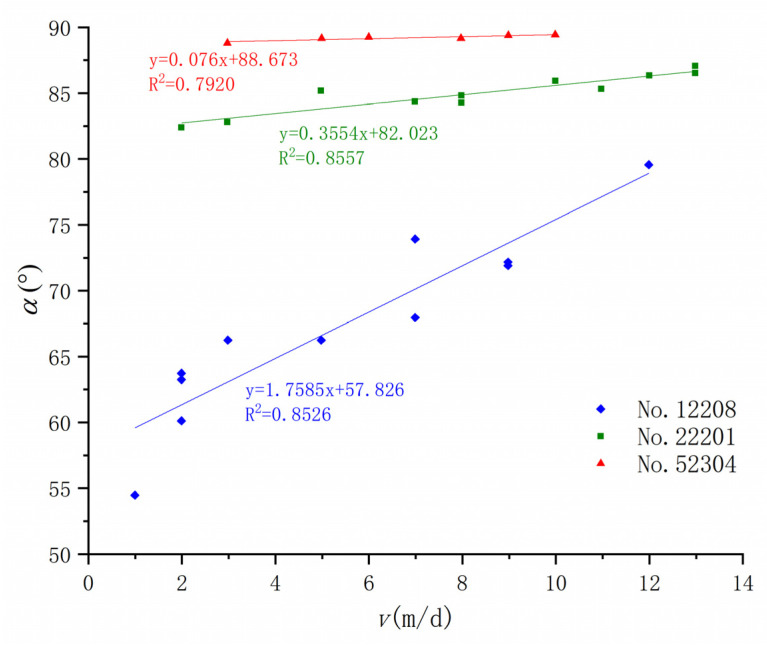
Relationship between ground fissure advance rate *v* and angle *α*.

We studied the relationship between the development of ground fissures and the mining speed caused by the mining of three working faces, 12204, 22201, and 52304, in the Daliuta Coal Mine, and found that the horizontal distance of the ground fissure caused by the mining of the three working faces is obviously positive, which verifies that these ground fissures formed before the advancing coal seam mining position. There is a negative linear relationship between *L* (Figure 9) and *T* (Figure 10) and the advance rate *v*, and a positive linear relationship between *α* and *v* (Figure 11). Therefore, the faster the coal seam is mined, the smaller the fracture distance, the larger the fracture angle, and the shorter the fracture development period.

#### 4.1.3. Comprehensive Parameters of Geological Mining Affecting the Development of Ground Fissures

Many studies have found that a larger ratio of bedrock thickness to mining height is more conducive to the development of ground fissures [54]. A smaller *v* also leads to more ground fissure development. Therefore, the two geological parameters of bedrock thickness (*H*) and mining height (*m*) are associated with *V*, and *K* is used as the comprehensive influence parameter of geology and mining:

(2) K=v∗h/m

Through the regression method, the relationship between *L*, α, and the comprehensive influence parameter *K* can be further defined as follows:

(3) L=−5.7234ln(K)+33.483

(4) α=8.0032ln(K)+45.590

This method couples the mining thickness ratio of the bedrock and the mining speed through the analysis of field–measured data. The calculation methods of the ground fissure distance and ground fissure angle under different burial depth conditions are given. The proposed comprehensive influencing parameters of geological mining provide a reference for the prediction of the development location of mining–induced ground fissures in different mining areas [55]. However, its applicability in other mining areas needs to be further verified. 

#### 4.1.4. Influence of Rock and Soil Structure and Characteristics on Ground Fissures

To accurately explain the law of surface subsidence, the overlying rock structure is divided into two different media: the surface loose layer and the rock layer. The impact on the development of ground fissures is divided into the following situations:

(1) The composition and structure of the overlying rock. When the rock and soil mass is thin or the bedrock contains hard rock, the structure and property of the rock and soil mass have a great influence on the surface settlement, and the rock and soil mass is not enough to digest the uneven settlement caused by rock fracture and movement, so the ground fissures are formed [56]. Li Chunyi et al. believe that when the key layer is broken [37], the shear failure of rock layer is easy to cause the ground fissure due to the thin loose layer (only 20 m) [37].
(2) Physical and mechanical properties of loose layers. The formation and properties of mining fractures are related to the existence of loose beds. The critical deformation value is related to the physical and mechanical properties of rock strata. If different physical and mechanical properties are subjected to the same additional mining stress, the stress–strain relationship is also different, resulting in different types and causes of surface fractures [57]. Liu Shouhua et al. demonstrated through simulation tests that the type and size of surface ground fissures are related to the properties of rock and soil mass [36]. For example, when clay is the same, clay with a high plasticity index and high viscosity will be affected by the fracture of bedrock and form the wider ground fissure, which are difficult to close after stress release. Tao Hailiang et al. [26] believed that the cohesion of sandy loam was small, so the critical deformation value of surface fractures induced by mining in sandy loam was smaller than that of clay. Due to the mutual influence of the cohesion and internal friction angle, the limit of fracture development depth in sandy loam soil is greater than that in clay soil [58]. In addition, vegetation roots enhanced the tensile strength of the shallow soil, resulting in a smaller shallow width of ground fissures and a bundle development in the vertical direction.
(3) Rock strength. After the mining of the working face, the overlying rock layer moves and bends in the form of beams or plates along the normal direction of the surface. When the moving deformation arrives to the surface, the surface’s tensile and curvature deformation exceed the tensile strength of the soil, so the ground fissure is created.. The smaller the tensile strength of the overlay is, the more likely it is to form ground fissures on the surface [58]. In addition, the roughness of the bedrock surface has a great influence on the overlying soil layer. When the surface is rough, the soil layer will produce obvious fissures, and ground fissures will easily form [59].

Moreover, the topography, surface movement deformation size, mining technology, overlying rock structure, and physical and mechanical characteristics of the loose layer will also have a certain impact on the development of mining ground fissures.

#### 4.1.5. The Effect of Fault Activation on the Drop of Ground Fissures

A fault is a structure in which rock strata or rock mass displacements obviously occur along the fracture plane. It is formed by rock strata fractures caused by crustal movement and relative movement at the fracture plane. Faults are relatively stable for short periods of time and they are not affected by external forces, and the amount of movement is too small to form ground fissures on the surface. When coal mining causes the overall movement of the overlying strata, the original stress balance is broken, which is bound to cause one side of the rock mass to slip suddenly along the sliding surface. Therefore, the surface collapse forms ground fissures [60,61].

The formation process and characteristics of such ground fissures are as follows: at first, it appears as surface ground fissuring and then develops into a step–like collapse with obvious surface drops on both sides of the ground fissure. The ground fissure width and depth are small, but the drop is large. The ground fissure strike is parallel to the fault strike and disappears at the surface outcrop of the fault [62].

The main reason for the collapse–type ground fissures caused by fault activation is the drop of the ground fissures. The calculation of the drop can generally be made up of two parts: the sinking value of the corresponding position in the absence of a fault and the vertical moving component produced by a fault slip. The surface subsidence value without faults can be predicted using the probability integral method [63]. The vertical component of a fault slip is formed by coupling geological and mining parameters such as the fault dip angle, mining thickness, and loose layer thickness with surface deformation and one or more empirical coefficients. The most commonly used empirical formula is as follows:

(5) $$\Delta W_{F}=\frac{m\times q\times H\times \sin\delta }{2000\left(0.1h+1\right)}$$

where $\Delta W_{F}$ is the vertical component of the fault slip; *m* is the mining thickness, 3.5 m; *q* is the subsidence coefficient, which is 0.65; *H* is the mining depth; *δ* is the fault dip angle; *h* is the thickness of the alluvial layer, 11.5 m.

(1) When the dip angle of the fault is less than 30° or the cross–sectional area of the mined coal seam (the product of the total thickness of the mined coal seam and the mined inclined length) is less than 2000 square meters, only ground fissures appear without steps on the surface [64]; when the cross–sectional area of the mined coal seam reaches 2000 square meters, a step will appear at the fault outcrop with a drop of 0.1. The relationship between the step drop and mining sectional area is as follows:

(6) ΔW=b·A

where ΔW is the drop of the sinking step; *b* is the empirical coefficient, and its value is usually 0.00005; *A* is the sectional area of the mined coal seam.

(2) The empirical formula for calculating the drop of the sinking steps is:

(7) $$\Delta W=\frac{K\cdot W_{m}\sqrt{\sin\delta }}{x/l}$$

where *K* is the fault influence coefficient; *δ* is the fault dip angle; *Wm* is the maximum subsidence value in the surface subsidence basin; *l* is the half basin length of the surface subsidence basin; *x* is the distance from the fault outcrop to the maximum subsidence point.

(3) The empirical formula for calculating the horizontal movement difference in the sinking step (i.e., the width of the step) is:

(8) $$\Delta U=\Delta W\cdot cotangent\left(\alpha_{F}\right)$$

where ΔU is the horizontal movement difference; cotangent is the sign of cotangent in trigonometric functions; $\alpha_{F}$ is the dip angle of the fault.

The advantages of this method are that the calculation process is simple, and it has good generalization in specific mining areas. However, the calculation accuracy mainly depends on the accuracy of the empirical parameters. For example, *a, b,* and *K,* which are used in the formula, are all empirical parameters.

### 4.2. Influence of Surface Deformation on the Size of the Ground Fissure

Mining–induced ground fissures are a form of surface damage, and the most common ones are extensional fissures developed in tensile surface areas. The larger the horizontal surface deformation, the larger the ground fissure width. Generally, if the surface is of large plastic clay, the horizontal deformation value of the ground fissure is 6–10 mm/m; for sandy clays with low plasticity, the horizontal deformation value of the fracture is 1–3 mm/m [65]. There is a positive linear correlation between the ground fissure width and tensile deformation. For example, in the Shendong mining area, the relationship between ground fissure width *W_L_* and horizontal surface deformation *ε* is as follows:

(9) $$W_{L}=0.2127\varepsilon +0.0323$$

where *W_L_* is the relationship between the ground fissure width; *ε* is the horizontal surface deformation. That is, when the horizontal deformation increases by 1 mm/m, the ground fissure width increases by 0.2127 m. The minimum ground fissure width detected on site is 0.045 m, and the horizontal surface deformation here is only 0.16 mm/m. The surface soil particle size of the mining area is 0.005–0.075 mm, which is a typical sandy silt with little plasticity. Therefore, surface tensile fractures are widely developed in the mining area, almost covering all mining faces. Sometimes, there will also be compression fractures in the surface compression region, and there is a logarithmic increasing relationship between the height of the fracture uplift *h_J_* and the horizontal compression deformation *ε*:

(10) $$h_{J}=0.1605\ln(\varepsilon )-0.2302$$

where *h_J_* is the height of the fracture uplift; *ε* is the horizontal surface deformation. In the Shendong mining area, when the compression deformation is greater than 5 mm/m, extrusion ground fissures begin to develop. With the increase in the compression deformation value, the ground fissure height increases gradually. When the compression deformation is 5–15 mm/m, the fracture height increases significantly. When the compression deformation is more than 15 mm/m, the fracture height is basically stable, being generally less than 0.3 m. 

Undoubtedly, there is a linear proportional relationship between the horizontal deformation of the surface and the width of the ground fissure; however, the key problem is that the physical and mechanical properties of different surface sediments are different, and the critical value of surface horizontal deformation that produces ground fissures, as well as the influence of the coefficient is different.

### 4.3. Influence of Topography on the Development Location of Ground Fissures

**Figure 12:**
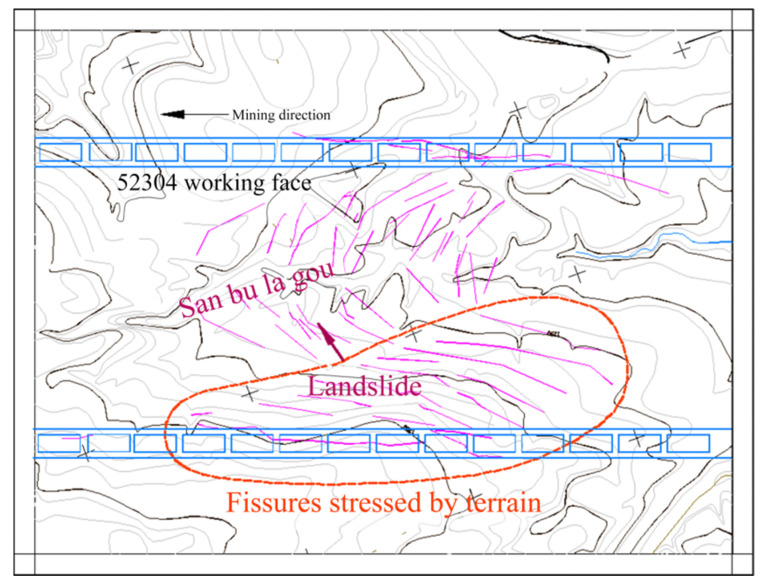
Mining ground fissures under valley topography.

When the surface above the working face is undulating, topographic factors further affect the size of surface deformation. For example, when mining in concave topography, the amount of surface subsidence decreases, while when mining in convex topography, the amount of surface subsidence increases [66]. This is caused by slope slippage caused by mining. The slippage of the slope body obviously stresses the ground fissures, and these are the sliding–type of ground fissures mentioned above. Factors such as the gully slope and relative position of the slope body and working face further change the development location and propagation direction of fractures. For example, the 52304 working face is 4547.6 m long and 301 m wide, the average coal seam thickness is 6.94 m, the coal seam inclination is 1~3°, and the average buried depth is 235.0 m. During the mining of the Sanbulagou of the 52304 working face of the Daliuta Coal Mine in the Shendong mining area, the development of ground fissures is obviously controlled by the direction of the mining–induced landslide, and presents a plane distribution that develops in parallel with terrain contour lines, as shown in Figure 12.

We used the UDEC discrete element software to carry out a computer numerical simulation of the change in the development location of ground fissures when the slope and location of the surface gully changed, respectively [57]. To analyze the development rules of different slopes of surface gullies, when the other conditions are identical, we calculated the ground fissure development rules under five conditions of gully slope: 10°, 20°, 30°, 40°, and 50°. To analyze the rule between the different gully positions and the ground fissure angles, six numerical models with the distance of the boundary of 0, 0.1D, 0.2D, 0.3D, 0.4D, and 0.5D (D is the working face dimension) were established. The results show that there is an obvious quadratic polynomial relationship between the fracture angle and gully slope. With the increase in the gully slope, the fracture angle decreases first and then increases. That is:

(11) $$\delta_{H}=0.0196\alpha^{2}-1.0976\alpha +89.84$$

where $\delta_{H}$ is the fracture angle; α is the gully slope. By solving the limit of Equation (11), we can obtain α=28.0°, that is, when the gully slope is 28.0°, the fracture angle is the minimum, and its value is $\delta_{H\min}=74.5{}^{\circ}$; when α=0, $\delta_{H}=89.84{}^{\circ}$; in other words, during flat mining, the slip component caused by the slope disappears and the ground fissure is of a collapse–type, and the collapse location is close to the boundary of the goaf, which further verifies the rationality of this formula.

There is a linear decreasing relationship between the gully valleys’ position and fracture angle. When the center of the gully valleys shifts from the boundary of the working face to the center of the working face, the fracture angle decreases linearly. That is:

(12) $$\delta_{H}=-64.3\frac{d}{D}+107.47$$

If we let $\delta_{H}=90$, we can obtain d=0.27D. That is, when the center of the gully valleys shifts 0.27 times the size of the working face boundary, the ground fissure angle $\delta_{H}=90{}^{\circ}$; at this point, the fracture development position is directly above the working face boundary. When d<0.27D, the location of the fracture development is inclined to one side of the coal pillar; when d>0.27D, the location of the fracture development is inclined to one side of the goaf.

The accuracy of the above research results is well verified by the field measurements of the Daliu Tower’s 52304 working face. The maximum error is 3.4% when the measured value of the ground fissure angle is compared with the calculated value.

Relevant studies show that topographic relief mainly affects the occurrence location of ground fissures. The main reason is the slope slip caused by mining; at the same time, it causes the change in the development position of the ground fissures [67]. The slope and location of the gully valleys are key factors affecting the location of ground fissures. However, how their internal stress field evolves still needs to be fully understood. If this is analyzed from the angle of slope stability, it remains to be seen whether we can obtain the same conclusion as the research results.

## 5. Formation Mechanism of Mining–Induced Ground Fissures

According to the above analysis, the formation process of mining–induced ground fissures is that the overlying rock is broken and the surface is deformed by coal mining; at the same time, the topsoil is pulled and formed. Ground fissures are just manifestations of the overall destruction of the rock and soil. When coal seams are mined, the breaking of rock strata controls the dynamic process of surface subsidence, and the loose layer plays the role of absorbing the uneven settlement [68]. Therefore, the thinner the loose layer, the more significant the characteristics of the non–continuous deformation on the surface, and the easier it is for the ground fissure to form. Tensile ground fissures are formed because the surface tensile deformation exceeds the tensile strength of the topsoil. The formation of collapse–type ground fissures is due to the load of the overburden strata exceeding the bearing capacity of key strata (thick hard strata), resulting in the overall cutting off of the overlying rock and soil. What makes it more complicated is that sliding fractures are formed by the comprehensive influence of slope slip and rock and soil fall caused by mining [69].

### 5.1. Formation Mechanism of Tensile and Compressive Fractures

**Figure 13:**
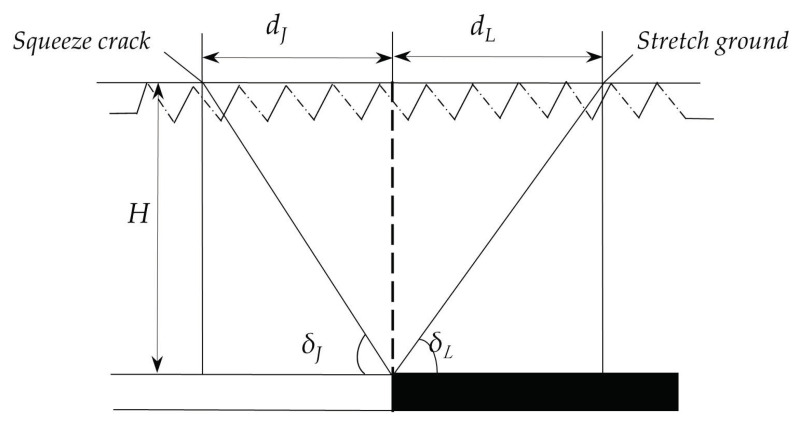
Tensile fracture and compression fracture.

Tensile and compressive ground fissures are generally caused by surface deformation. This type of the ground fissure gradually extends down from the surface, and it is reasonable to use surface horizontal deformation to determine whether the ground fissures formed. Affected by mining, within the influence range of surface subsidence, there are tensile deformation zones and compression deformation zones; the demarcation point is located at the inflection point of the surface deformation [70]. During the process of advancing the working face, tensile fractures generally develop in the tensile zone ahead of the working face, and compression fractures generally lag behind the working face and develop in the compression zone. The horizontal distance between the tensile fracture and the advancing position of the working face is called the tensile fracture advance distance, denoted as *d_L_*; the angle between the line between the fracture and the advancing position of the working face and the horizontal line is called the leading angle of the tensile fracture, denoted as $\delta_{L}$. The horizontal distance between the extrusion–type ground fissure and the advancing position of the working face is called the extrusion–type ground fissure lag distance, denoted as $d_{J}$; the angle between the line connecting the ground fissure and the advancing position of the working face and the horizontal line is called the lag angle of the extrusion ground fissure, denoted as $\delta_{J}$; the mining depth of the fracture development location is denoted as H (unit m), as shown in Figure 13.

The horizontal deformation ε(x,y,ϕ) at any point on the Earth’s surface along any direction ϕ is:

(13) $$\varepsilon (x,y,\phi )=\frac{1}{W_{0}}\left\{\varepsilon^{0}(x)W^{0}(y)\cos^{2}\phi +\varepsilon^{0}(y)W^{0}(x)\sin^{2}\phi +\left[U^{0}(x)i^{0}(y)+U^{0}(y)i^{0}(x)\right]\sin\phi \cos\phi \right\}$$

where $W_{0}$ is the maximum surface subsidence value when both the strike and inclination directions reach full mining; $W^{0}(x),\varepsilon^{0}\left(x\right),U^{0}\left(x\right),i^{0}(x)$ is the subsidence value, horizontal deformation value, horizontal movement value, and tilt value of the point with abscissa *x* on the main section when the inclination direction reaches full mining; $W^{0}(y),\varepsilon^{0}\left(y\right),U^{0}\left(y\right),i^{0}(y)$ is the subsidence value, horizontal deformation value, horizontal movement value, and tilt value of the point with abscissa y on the inclined main section when the strike direction reaches full mining.

Assuming that the tensile modulus of the surface soil is $E_{t}$, the compressive modulus is $E_{s}$, the tensile strength is $\sigma_{\mathrm{pull}}$, and the compressive strength is $\sigma_{\mathrm{press}}$, then the ultimate tensile strain and compressive strain of the soil are:

(14) $$\varepsilon_{\mathrm{pull}}=\frac{\sigma_{\mathrm{pull}}}{E_{t}},\;\varepsilon_{\mathrm{press}}=\frac{\sigma_{\mathrm{press}}}{E_{s}}$$

That is, the horizontal deformation value of the surface somewhere is ε (ε>0 is tensile deformation, ε<0 is compressive deformation); if $\varepsilon >\varepsilon_{\mathrm{pull}}$ or $\left|\varepsilon \right|>\left|\varepsilon_{\mathrm{press}}\right|$, the surface will produce tensile or extrusion ground fissures.

### 5.2. Formation Mechanism of Collapse–Type Fractures

Collapse fractures are the result of the joint action of rock strata breaking and surface subsidence. They are usually manifested as a step sinking subsidence of the Earth’s surface, being especially common in the western shallow coal seam mining area. When the rock fragmentation is small or the thickness of the loose layer is large enough, rock strata fracture has relatively little influence on surface collapse–type ground fissures. Related studies have shown that the dynamic development of a collapse fracture is similar to the periodic fracture of rock strata; its plane distribution presents a “C”–shaped feature similar to the “O”–shaped ring on the basic top [71,72]. Therefore, it is reasonable to reveal the formation mechanism of collapse fractures using the theory of rock strata breakage.

According to rock fracture theory, the fracture of the basic roof will cause a violent movement of the overlying rock and surface. In longwall mining, the basic roof is regarded as a thin plate, which better explains the periodic fracture of rock strata. Before the first break of the basic top, it can be regarded as a thin plate for the four sides of the fixed support. As the working face advances, after reaching the initial breaking step of the basic top, the top plate behind the working surface is constantly destroyed to form a goaf, the rear of the basic roof can be regarded as a simply supported condition, and the other three sides are still the plate with a fixed support condition [73]. From this, the calculation method of the first break–up and the cycle break–up steps of the basic top is:

(15) $$a_{1}=\frac{h}{1-\mu^{2}}\cdot \sqrt{\frac{2\sigma_{s}}{q}\cdot \frac{1+\lambda^{4}}{1+\mu \lambda^{4}}}$$

(16) $$a_{2}=\frac{2h}{1-\mu^{2}}\cdot \sqrt{\frac{\sigma_{s}}{q}\cdot \frac{2+\lambda^{4}}{4+3\mu \lambda^{2}}}$$

where h is the basic roof thickness, m; μ is the Poisson’s ratio; $\sigma_{s}$ is the tensile strength, MPa; q is the own gravity and the overlying load, MPa; λ=a/b is the geometric shape coefficient of the goaf.

When the length of the goaf reaches the basic roof breaking distance, the basic roof breaks for the first time, and the first collapse–type ground fissure will appear on the surface. Therefore, it can be considered that collapse fractures are synchronous with basic roof fractures.

**Figure 14:**
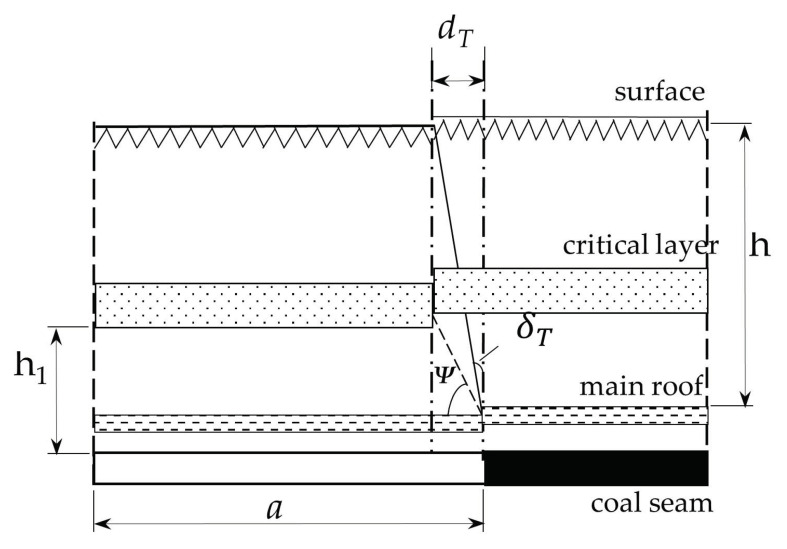
Comparison of upper and lower wells with collapsed fractured wells.

However, if there is a critical layer above the basic top, with the advance of the working face, when the advance distance A reaches the breaking distance of the key layer, the first breaking of the key layer will occur, all the overlying strata will collapse, and the first collapse–type ground fissure will appear on the surface [74]. Considering the influence of rock fracture angle ψ, the location of the surface collapse fracture lags behind the working face mining for a certain distance, and this is called collapse–type ground fissure lag distance, denoted as $d_{T}$. The vertical angle of the line between the location of the surface collapse–type ground fissure and the mining location of the working face is called the lag angle of the collapse–type ground fissure, denoted as $\delta_{T}$, as shown in Figure 14. The fracture of the critical layer generally lags behind that of the basic roof. Therefore, the position of the surface collapse fracture lags behind the mining position of the working face, and the fracture spacing is negative. When there are multiple formations in the overlying strata, the rock layer that completely or locally controls the activity in the rock mass is called the critical layer, that is, the main bearing layer. The form of “plate” or “beam” structures can bear the partial weight of the upper rock layer before breaking; after the fracture, the masonry beam structure is formed, and its form is the rock layer movement. The main basis of the critical layer discrimination is its deformation and breaking characteristics. When the key layer is broken, the sinking deformation of the upper rock layer is coordinated with each other. 

The collapse fracture distance $d_{T}$ can be calculated using the following formula:

(17) $$d_{T}=\frac{h_{1}}{\tan\psi }$$

where $h_{1}$ is the distance between the key layer and the basic top, m; ψ is the fracture angle of the strata.

The lag angle $\delta_{T}$ of a collapse fracture can be calculated by the following formula:

(18) $$\delta_{T}=\tan^{-1}\left(\frac{d_{T}}{h}\right)=\tan^{-1}\left(\frac{h_{1}}{h\tan\psi }\right)$$

where *h* is the distance from the ground surface to the basic roof, m.

As can be seen from the above equation, the lag distance of the collapse fracture is proportional to the distance between the key stratum and the basic roof. The tangent of the lag angle is proportional to the ratio of the distance from the critical layer to the basic top and the distance from the surface to the basic top.

### 5.3. Mechanical Mechanism of Sliding Fractures

The generation of sliding ground fissures is closely related to the slip of the surface slope. The change in the stress field forms the basis for judging slope stability. Therefore, combining slope stability theory with mining subsidence theory can reveal the mechanical mechanism of sliding fractures.

**Figure 15:**
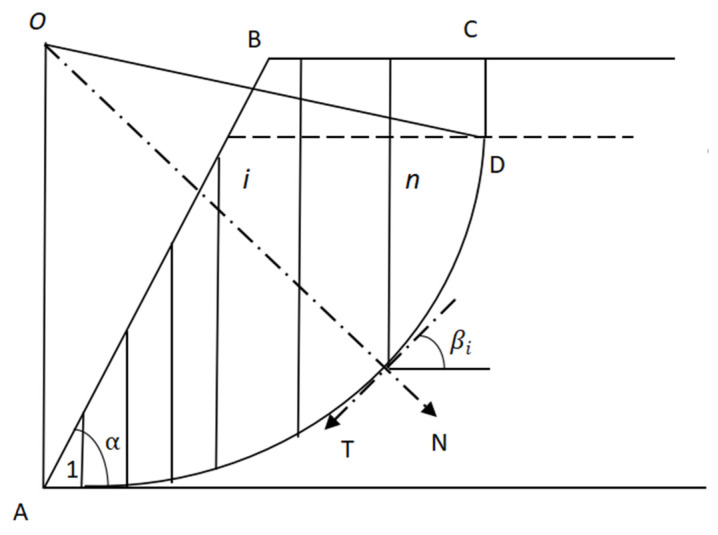
The calculation model of the slicing method.

The basic steps are as follows: (1) Analyze the forces on the slope body. When the overburden is damaged by mining, the overlying slope is not only affected by its own gravity, but also by the additional stress of mining, thus changing the overall stress state of the slope. Various mining–influenced stresses can be decomposed into three additional forces: additional horizontal component $F_{\varepsilon }$, additional shear force $F_{\tau }$, and additional vertical tension $F_{\omega }$. Then, the additional stress of mining and the gravity of the slope itself should be superimposed. (2) Determine the most dangerous sliding surface of the slope. It is generally believed that there is a potential arc–shaped sliding surface in the soil slope, and the sliding surface position can be determined according to the limit equilibrium theory of loose media. (3) Calculate the slope stability. The strip division method is adopted, that is, the sliding body is divided into *n* strip blocks along the vertical direction, and various stresses on each strip block are projected to the sliding surface. Then, it is decomposed into tangential force *T* along the sliding surface and normal force *N* perpendicular to the sliding surface, as shown in Figure 15. In the figure, *AB* is the natural surface of the slope, *AD* is the circular sliding surface, α is the average slope inclination, and $\beta_{i}$ is the sliding surface inclination of the *i* strip. 

The tangential force pointing downhill along the sliding surface is also called the sliding force, the force pointing uphill is also called the anti–slip force, and the anti–skating force can be obtained from the normal force. When the sliding force is less than the anti–sliding force, the slope is stable; on the contrary, slope slip will occur. (4) Identify the location of ground fissures. From the valley of the slope, along the uphill direction of the slope, calculate the sliding force and the anti–sliding force of each strip one by one, and add them up one by one; take the ratio denoted as *K*, as shown in Equation (19).

(19) $$K=\frac{\sum_{i=1}^{n}T_{i}}{\sum_{i=1}^{n}S_{i}}=\frac{\sum_{i=1}^{n}\left(G\left\{(1+\eta )\sin\beta_{i}+P\left[\lambda (\varepsilon +\varepsilon^{\prime })+\xi (i+i^{\prime })\right]\cos\beta_{i}\right\}+\eta CL\sin\beta_{i}\right)}{\sum_{i=1}^{n}\left(G\left\{(1+\eta )\cos\beta_{i}-P\left[\lambda (\varepsilon +\varepsilon^{\prime })+\xi (i+i^{\prime })\right]\sin\beta_{i}\right\}\tan\phi -\eta CL\cos\beta_{i}\tan\phi +(1-\eta )CL\right)}$$

Among them, the main parameters involved are (1) mining strength coefficient *P*, and its value is $P=\frac{mD\tan\alpha }{H_{0}F}$, *m* is the mining thickness, m; *D* is the working face width, m; α is the average dip angle of the slope, (°); *H_0_* is the average mining depth of the coal seam under the slope body, m; *F* is the slope rock–soil layer coefficient. (2) Settlement disturbance coefficient η; its value is: $\eta =\frac{PW}{H_{0}-h}$, where *W* is the subsidence value of the mining slope top and *h* is the height of the slope body. (3) Mining subsidence parameters. ε and i, respectively, represent the maximum static horizontal tensile deformation and the incline deformation on the upslope surface along the main section of the slope incline after settling, mm/m; $\varepsilon^{\prime }$ and $i^{\prime }$ are the dynamic horizontal tensile deformation and the inclined deformation in the mining process, respectively. (4) Physical and mechanical parameters of the slope body. *G* is the slope weight force; λ is the lateral pressure coefficient of the slope rock and soil layer, and its value is: $\lambda =\frac{\mu }{1-\mu }$, *μ* is the Poisson’s ratio, ϕ is the internal friction angle of the slope, and $\beta_{i}$ is the inclination angle of the sliding surface of the bar, i; *C* is the cohesion; *L* is the length of the sliding surface, m; ξ is the ration of the slope height to the mining depth.

According to the limit equilibrium theory, when *K* = 1, without considering the effect of the upper side strip, the slope body will produce the first slip here, and form the first sliding ground fissure; if the stress of the upper strip accumulates, *K* keeps going up, indicating that the stability of the upper slope body becomes worse. At this point, the lower strip can be regarded as a continuous slope, and when the value of *K* is the maximum, the stability of the slope here is the worst, and the slope body will be partially fractured here, thus producing a sliding–type of ground fissure [75].

**Figure 16:**
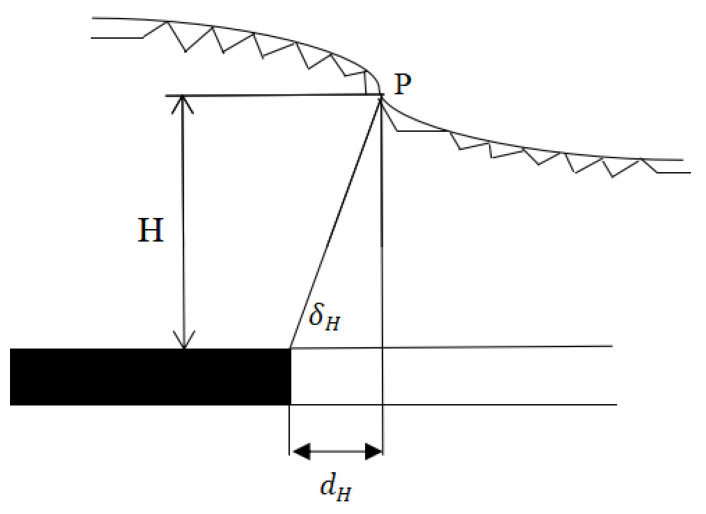
Schematic diagram of the sliding ground fissures.

This method combines mining subsidence theory and slope stability theory, and also combines the influence of mining with the physical and mechanical properties of the slope through stress analysis; therefore, the quantitative calculation of sliding ground fissures can be achieved, as shown in Figure 16. $\delta_{H}$ is the sliding fracture angle. In the 52304 working face of the Shendong mining area, the calculation of the fracture angle using this method has been well verified. The calculated values of the sliding fracture angles on both sides of the gully valleys are 47.3° and 75.3°, respectively, and the measured values are 46.7° and 76.4°, respectively, showing high accuracy.

## 6. How to Treat Mining–Induced Ground Fissures

Ground fissures caused by mining can be divided into two: permanent ground fissures and temporary ground fissures. Permanent ground fissures cause irreversible damage to the ecological environment, and temporary ground fissures in the process of mining threaten the safety of underground mining. There is no doubt that permanent ground fissures cause great harm to the surface ecological environment, and from the point of view of environmental protection, such ground fissures must be managed. However, temporary ground fissures caused by mining have a certain self–healing ability [76]. The question remains regarding whether they too need to be managed or governed.

### 6.1. Treatment Standards for the Temporary Ground Fissure

Regarding the temporary ground fissure, some scholars have proposed to treat them after the surface subsidence stabilizes. However, once the upward–moving rock fracture zone makes contact with the downward ground fissures, it will endanger the safety of underground mining, and some water may come up and sand bursts and air leakage may occur. Therefore, the basis for managing ground fissures must be provided. Simply speaking, whether the rock fissures and ground fissures are connected forms the basis for managing temporary ground fissures. In other words, based on whether the sum of the development height of the water–conducting fracture zone and the development depth of the ground fissure is greater than the mining depth, whether the ground fissure needs to be treated can be judged [77]. It is found that there is a direct proportional linear relationship between the fracture depth and width, and a logarithmic increasing relationship between the depth and height. We have studied the relationship among the fracture width, drop, and depth in the Shendong mining area, namely:

(20) d=11.380W+0.916 

(21) d=1.5156ln(h)+5.7372

Because fracture depth detection is more complex, and the fracture width and drop are relatively easy to measure, the maximum safety width of the ground fissure or the maximum safety drop can be used to judge whether the ground fissure needs to be treated.

### 6.2. Governance Methods

**Figure 17:**
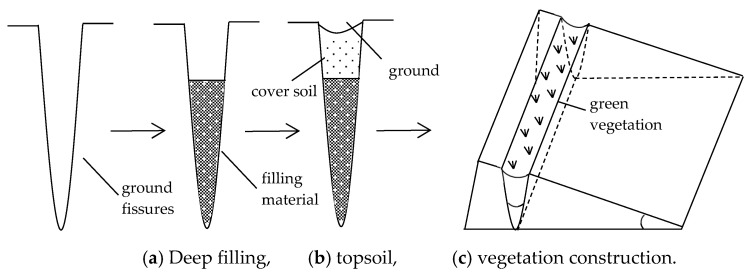
“Three–step method” for treating ground fissure.

The treatment of ground fissures is generally divided into three steps: deep filling, shallow overburden soil, and vegetation construction. First, grout is injected into the deep part of the fracture and filled to about 0.5 m above the surface. The filling grout can be mud, fly ash, paste, and other materials. Then, after the slurry is solidified, the consolidated body in the ground fissure is covered with soil and compacted, and an arc ground fissure groove is constructed on the upper surface of the covered soil [78]. Finally, vegetation construction is carried out in the fissure groove according to the ecological environment characteristics and the current situation of the control area; grasses or shrubs with strong drought resistance may be selected, as shown in Figure 17.

The stability of the filling body and the suitability of the vegetation growth are key to the success or failure of the treatment of ground fissures [79]. Only when the depth is densely filled will the fracture not undergo secondary collapse. The type of vegetation needs to be appropriate to ensure the survival of the vegetation and improve the efficiency of the ecological restoration in the mining area.

## 7. Outstanding Issues

### 7.1. Observation Method

Ground fissure monitoring mainly includes monitoring the development position, length, width, drop, and depth of fractures. At present, the main technical means include field observation, remote sensing interpretation, laboratory simulation, etc. 

Field observation is the most effective and direct method to obtain the characteristics of ground fissure development; however, mining–induced ground fissures have the characteristics of a short development time and concealment, and, therefore, the intensity of fieldwork is high and the efficiency is low, while depth detection is more difficult [80]. A reasonable determination of the monitoring period is the key problem to be solved when conducting field observations. 

The advantage of remote sensing interpretation is that it can be used to quickly obtain a large range of ground fissure development information, but only the plane distribution characteristics of the ground fissures can be obtained. Remote sensing is also affected by the transit cycle of satellites, resulting in a loss of ground fissure development information between two monitoring periods.

Laboratory simulation includes two methods, similar material simulation and computer numerical simulation, and these can effectively solve the problem of the spatial and temporal acquisition of ground fissure information; these simulations can also be used to model fissures according to different geological mining conditions, and, therefore, they are becoming increasingly more popular. However, laboratory simulations must be validated with field tests. To sum up, depending on the different purposes of studies, ground fissures at the scale of the working face can be studied by combining the on–site monitoring station date and laboratory simulations [81]. 

Regardless of the observation method, attention should be paid to geological mining conditions such as the mining depth, overburden structure, working face layout, mining speed, and the type of developed ground fissures. In addition, a reasonable monitoring period needs to be determined to accurately uncover the dynamic development of ground fissures.

### 7.2. Dynamic Development Law of Ground Fissures

Most researchers believe that mining–induced ground fissures are part of a dynamic cycle of “ground fissuring–expanding–closing”. However, through high–density field monitoring, Hu Zhenqi found that part of the ground fissures in the 12406 working face of the Shendong Mining area showed two dynamic characteristics of “ground fissure–closure”, the fracture development period was 18 d, including two “ground fissuring–closing” processes with approximately equal duration. Whether this is a unique feature of a certain mining area or part of details that have not yet been discovered by other studies is unknown [82]. Since there is no mechanical explanation for the multi–period development of dynamic fractures, further research should be carried out from the following aspects to fully reveal the dynamic development of mining–induced ground fissures. ① Shorten the field monitoring period. For the key monitoring of ground fissures, continuous observation can be carried out in 1 day or less to avoid missing the dynamic development law of surface deformation and ground fissure characteristics in the active period. ② More detailed laboratory simulation experiments are an effective means. Combined with the excavation speed of the coal seam, all–weather rock and soil deformation monitoring should be carried out, which can reveal the synchronous development of the ground fissure, rock and soil layer deformation, and stress field evolution. ③ The dynamic change in the friction between rock strata and topsoil should be quantitatively studied [83].

### 7.3. Influencing Factors

In this study, the influencing factors of mining–induced ground fissures are classified into three categories: geological and mining conditions, surface deformation, and landform. Many studies have revealed the influence of a single factor on the development characteristics of ground fissures. In some studies, coupling several factors through mechanical analysis, the established prediction model better reveals the development process of ground fissures under specific conditions [84]. However, ① some key influencing parameters are still not perfect. For example, the mining strength coefficient refers to the damage degree of mining to rock and soil masses, which is estimated by the mining conditions and the empirical formula of the overburden lithology coefficient; its applicability under mining conditions of shallow buried coal seams needs further study. ② The coupling effect of mining factors and topographic factors on the development of ground fissures should be paid more attention to. On the one hand, underground mining causes the topsoil to break and form the ground fissure; on the other hand, the slip control effect of topographic factors on the development of ground fissures cannot be ignored, especially the stress effect of the slope, height, relative position of the slope body, and the working face on the fracture development location and propagation direction. The main way to solve the problem is, first, through correlation analysis to reveal the correlation between the development of ground fissures and other influencing factors; this will make clear the main controlling factors of mining–induced ground fissures [85]. Then, a scientific and reasonable mechanical explanation should be explored, and the mechanical triggering conditions of the ground fissure caused by the superposition of additional mining stress and soil load can be studied; this can reveal the coupling effects of mining disturbance factors and topographic stress factors on ground fissures.

### 7.4. The Mechanical Nature of Ground Fissures

Mining–induced ground fissures are the coupling result of rock fracture and topsoil deformation, but rock layers and loose–layer soil generally have obvious differences in their physical and mechanical properties. In fact, rock and soil mass is a complex structure of “layer, block, and medium”. Existing studies are based on certain simplified assumptions, which are not consistent with the actual situation. ① The probability integral method used in most studies is based on random medium theory [86]. However, the constitutive equation of this theory is unknown, and, therefore, the stress and strain cannot be studied uniformly, which leads to a failure in reflecting the mechanical behavior of “stress balance–mining disturbance–stress rebalancing” during mining subsidence. Therefore, rock strata movement and surface subsidence cannot be explained using a mechanical mechanism alone. ② Mohr–Coulomb strength theory oversimplifies the tensile stress section and was originally used to study soil shear failure. Therefore, it is not suitable to use this theory to study the tensile failure of soil. ③ The rock and soil mass overlying goaf is composed of discontinuous mediums and continuous mediums; there are essential differences in the mechanical behaviors of the two media, and few studies have considered the coupling effect caused by such essential differences in the formation mechanism of ground fissures. ④ Some studies often use a variety of abstract mechanical theoretical models. Is there any conflict between the models? Is it verified in combination with the actual situation? Often no instructions are given [87]. Therefore, the stress–strain evolution mechanism between different rock and soil layers is the core of the formation process of mining–induced ground fissures, and further rigorous theoretical research is necessary.

### 7.5. Ground Fissure Treatment

As can be seen from the above research, when a mining–induced ground fissure is connected with a rock fissure, to ensure the safety of underground mining, it is necessary to treat the temporary ground fissure during mining. Conventional burials and fillings are the main methods. However, the permanent ground fissure after surface settlement are treated in three steps: deep filling, shallow soil covering, and vegetation reconstruction [88]. It should be pointed out that, from the perspective of surface ecological environment protection, ground fissure governance must follow the following principles. ① Follow nature and adjust the measures to the local conditions. Fully consider the terrain and geomorphic form of the surface of the working face and the distribution of the ecological environment, and strictly abide by the laws of the local ecosystem, optimize the layout, and control ground fissures treatment technology which is proposed according to local conditions. ② Guiding and sustainable self–healing. The dynamic development of ground fissures with the advance of the working face should be fully considered. According to the relationship between the fracture development depth, width, and fall and working face mining progress, and the subsequent mining plan, one should make full use of the law of surface collapse and fracture development and avoid secondary treatment. ③ Economic and reasonable and easy to promote. When treating temporary ground fissures in the mining process, one must consider the treatment cost and the prospect of popularization [89]. The treatment method adopted should not only fully ensure the safety of underground mining, but also achieve perfect function, obvious effect, and reasonable economy, so that it has good operability and application prospects and achieves the goal of ecological, economic, and social benefits coordination.

## 8. Conclusions

(1) Mining–induced ground fissures are a kind of geological disaster caused by underground coal mining, especially during shallow coal seam mining. When the coal seam is buried shallowly, the water–conducting fracture zone of the rock can reach the surface, and the surface above the goaf can form a collapse fracture with the periodic breaking of the basic roof. When the coal seam is buried deeply, tensile ground fissures are generally formed by surface deformation, leading to the breaking of the topsoil. If mining is carried out under gully valleys, the slip and fracture of the mining slope will further change the development location of fractures [29,90,91].
(2) The development of mining–induced ground fissures has the following general characteristics: The temporary fractures in the process of mining show a “C”–shaped plane distribution, and the fracture range is close to or slightly beyond the boundary of the goaf. They initially appear on the surface above the center of the panel, perpendicular to the direction of advance, and then extend to the goaf boundary. The ground fissure is the widest and deepest in the center and gradually becomes narrower and shallower as it approaches the boundary. The average fracture spacing is consistent with the periodic breaking step of the basic roof. With the advance of the working face, the width and drop of the rear fracture gradually decrease and heal after the surface subsidence stabilizes [92,93]. At the same time, this development process is repeated in the fracture front; after mining, the ground fissures at the boundary of the goaf exist permanently, presenting an oval distribution.
(3) The main influencing factors of mining–induced ground fissures include geological mining conditions, surface deformation, and landform. Among them, the smaller the bedrock mining thickness ratio, the more complete the fracture development, the faster the coal seam mining, the smaller the fracture distance, the larger the fracture angle, and the shorter the fracture development period [94]. When the loose layer is thin or the overburden tensile strength is small, it is easier to form the ground fissure. The size of the ground fissures is related to the overlying soil, and clay with a high plastic index and a high viscosity easily forms large–width ground fissures. Surface deformation mainly affects the size of ground fissures, and there is a direct linear–ratio relationship between the ground fissure width and horizontal surface deformation [95]. Topography and geomorphology mainly affect the location of fracture development. There is a quadratic polynomial relationship between the fracture angle and gully valley slope, which decreases first and then increases [96]. There is a linear decreasing relationship between the fracture angle and gully valley position. When the gully valleys’ center gradually shifts from the working face’s boundary to the working face’s center, the fracture angle gradually decreases.
(4) Tensile fractures generally develop in the surface tensile deformation zone ahead of the mining position of the working face, and occasionally protruding compressive fractures develop in the surface compressive deformation zone. The collapse–type fracture is synchronous with the basic roof fracture, and the fracture spacing is equal to the periodic fracture step of the basic roof. However, if there is a key stratum above the basic roof, the position of the collapse fracture lags behind the mining position of the working face, and the lag distance is proportional to the distance between the key stratum and the basic roof [97,98]. The development location of the sliding ground fissures caused by mining under gully valleys can be judged by the ratio of the sliding force to the anti–sliding force on the sliding surface. When the ratio *K* (*K* represents the ratio of the sliding force and sliding resistance force of the slope body stress on the sliding surface) is the maximum, ground fissures occur.
(5) Scientific treatment methods should be adopted for ground fissures. To ensure the safety of underground production, when the sum of the local fracture depth and the water–conducting fracture zone is greater than the buried depth, the temporary fracture in the mining process must be treated [99]. The basis of judgment is the maximum safe width or drop of the ground fissure. To protect the surface ecological environment, the management of ground fissures should be based on the principle of adjusting the measures to local conditions and following nature; the management of ground fissures usually follows the three steps of deep filling, shallow covering soil, and vegetation reconstruction.
(6) Due to the coupling influence of different geological and mining conditions and topographic and geomorphic environments, the development of mining–induced ground fissures is complicated. The results of this article make up for the deficiencies of the relevant research, provide the basis and direction for future research, and have universal applicability and scientific guiding significance.
(7) In future research, the physical and mechanical properties of rock and soil, the evolution of stress fields, and the stress conditions generated by ground fissures should be focused on [100]. In addition, the process of rock fracture and topsoil deformation, especially the stress–strain mechanism of the interface between the bedrock and the loose layer, the formation mechanism of dynamic ground fissures, the construction of mechanical models considering the effect of multi–factor coupling, the comprehensive application of multi–technical means, and high–intensity mining effects on the formation mechanism of ground fissures caused by new coal mining technologies should be further studied.

## Figures and Tables

**Table 1:** Statistics of mining–induced ground fissures in China.

**(a) Basic Conditions of Ground Fissures In Mines.**						
**Number**	**Time**	**Province**	**Mine**	**Terrain**	**Forming Reasons**	**Data Resource**
1	1975	Shanxi	Horse Ridge Mine	Mountains	Rock break	WuLixin [21]
2	1994	Shandong	Xinglongzhuang Mine	Plain	Surface deformation	LiLiang [22]
3	1999	Henan	Guozhuang Coal Mine	Plain	Fault activation	Zhou Quanjie [23]
4	2001	Shaanxi	Dayan Kiln Mine	Loess gully	Rock fracture, deformation, slope slip	Fan Limin [4]
5	2008	Shanxi	Wuyang Mine	Hills	Surface deformation	Teng Yonghai [24]
6	2009	Inner Mongolia	Fengshuigou Coal Mine	Loess gully	Slope slip, surface deformation	Zhao Bingchao [25]
7	2010	Anhui	Huainan Mine	Plain	Surface deformation, Rock fracture	Hu Qingfeng [3]
8	2011	Henan	Pingdingshan Thirteen Mine	Plain	Surface deformation	Tao Hailiang [26]
9	2011	Shaanxi	Tongchuan mining area	Mountains	Rock break, slope slip	Yuantao [27] Tang Fuquan [16]
10	2012	Shanxi	Shuozhou mining area	Mountains	Surface deformation, slope slip	Xu Naizhong [28]
11	2012	Shaanxi	Shendong Daliu Pagoda	Loess Plateau	Rock cover break	Liu Hui [15]
12	2012	Shaanxi	Shendong Daliu Pagoda	Loess Plateau	Surface deformation	
13	2012	Shaanxi	Shendong Daliu Pagoda	Loess gully	Surface deformation, slope slip	
14	2014	Shaanxi	Binchang mining area	Loess Plateau	Surface deformation	Tang Fuquan [29]
15	2014	Shanxi	Hezhai Coal Mine	Hills	Rock break	Zhang Wenjing [30]
16	2015	Hebei	Shanhou Mine	Plain	Surface deformation	Zhangfeng [31]
17	2015	Shanxi	Dongpo Coal Mine	Mountains	Surface deformation, slope slip	Gaochao [32]
18	2015	Gansu	Huating mining area	Loess Plateau	Rock break	Dao Naiqin [33]
19	2016	Liaoning	Xiaonan Coal Mine	Plain	Rock fracture, fault activation	Yangfan [34]
20	2016	Guizhou	Jie Niang Ping	Mountains	Slope slip, fault activation	Shi Wenbing [35]
21	2018	Shaanxi	Ningtiaota Coal Mine	Hills	Surface deformation, slope slip	Zhao Bingchao [25]
**(b) Topographic Parameters of Mine Ground Fissures.**						
**Number**	**Panel**	**Mining Depth (m)**	**Coal Seam Thickness (m)**	**Maximum Width of Ground Fissure (m)**	**Maximum Height Difference of Ground Fissure (m)**	**Maximum Depth of Ground Fissure (m)**
1	402	115	6	4	0.7	
2	4314	325	8.8	0.3	0.5	3.4
3	2228	139	3.7	0.63	0.65	
4		172	3.5–5.0	2	0.73	5
5	7511	270	6.49	0.3	0.3	
6		220	52.2	0.6	15	
7	8104	460	9.75	0.5	0.6	
8	11022	315	5.8	0.5		3.83
9	D508	180	2.4	1	1.9	
10		265	14.4	0.15	1.5	
11	12208	40	7.4	0.3	0.55	
12	22201	72	3.9	0.42	0.49	5.8
13	52304	235	6.9	0.42	1.2	6
14	B40301	350	8	0.1		5.6
15	2212	160	2.5	0.9	0.2	
16	6208	360	3.2	0.2	0.05	
17	914	265	14.4	0.71		9.84
18		500	22	5	4	66
19	W2–705	400	2.8	6		>10
20	A9	120	3.0	9.5	3.2	
21	N1206	162	5.9	0.8	1.2	

## Data Availability

The data used to support the findings of this study are available from the corresponding author upon request. The data is uploaded to the journal system in the form of attachments.
